# Volar Radiocarpal Dislocation: A Case Report and Review of Literature

**DOI:** 10.7759/cureus.9091

**Published:** 2020-07-09

**Authors:** Mohammed Sabr, Hosam T Mashrah, Abdulaziz H Abed, Hamdi Arabi, Fawaz Pullishery

**Affiliations:** 1 Orthopedics, National Guard Hospital, Al-Madinah, SAU; 2 Medicine and Surgery, Taif University, Taif, SAU; 3 Medicine and Surgery, Alfaisal University College of Medicine, Riyadh, SAU; 4 Community Dentistry and Research, Batterjee Medical College, Jeddah, SAU

**Keywords:** volar radiocarpal dislocation, palmar radiocarpal dislocation, radiocarpal dislocation

## Abstract

Radiocarpal dislocations (RCDs) are one of the rare injuries that happen to the wrist in which there is a partial or complete loss of contact between the carpus and distal radius. We present the case of volar RCD in a 25-year-old male patient. He reported to the ED with pain on the wrist of his left forearm. The patient had met with a motor vehicle accident and was put on forearm cast in the previous hospital. Initial clinical examination showed swelling with no visible deformity with good capillary fill; X-ray images showed no fracture, and he was again put on forearm cast giving an orthopedic clinic appointment. A missed diagnosis of left wrist complete volar RCD was found when we reviewed the X-ray, and the patient was called for immediate surgical treatment. He was treated under general anesthesia with closed reduction, and three parallel percutaneous Kirschner wires were pinned to the left radiocarpal joint. Occupational therapy to improve the range of motion and muscle strengthening were done, and clinical follow-up showed improvement in the extension, flexion, and muscle power. The patient was satisfied with the outcome and after three months of follow-up showed no new problems.

## Introduction

Radiocarpal dislocations (RCDs) are considered to be one rare type of carpal dislocations. In this type of injury, there is a dislocation of the radiocarpal joint due to partial or complete loss of contact between the carpus and dorsal distal radius that causes a complex ligamentous injury to the carpal instability complex of the wrist [1]. Reports show that the incidence of this injury is rare, which accounts for 0.2% of all dislocations [2-3]. According to Moneim et al., the prevalence of this injury is about 20% among all wrist injuries [4]. Literature shows that only a few case series have reported cases that included more than 10 RCDs, a case series presented by Dumontier et al. that included 27 RCDs was the maximum reported so far [5]. Most of the RCDs are reported in young males following high impact traumatic injury to the wrist joint from motor vehicle collisions [6-8].

In this article, we describe the presentation and surgical management of complete palmar RCD in the left wrist from a motor accident with five months of postsurgical follow-up.

## Case presentation

A 25-year-old right-handed electrical technician medically free, came to our ED in Prince Mohammed Bin Abdulaziz National Guard Hospital in Al-Madinah city one week after admission in another hospital because of short time. He had loss of consciousness with head trauma, postmotor vehicle accident. He was the driver using seat belt without a history of ejection from the car. He complained of pain on the left distal forearm, which was on below elbow splint, which was then removed. On examination: there was visible swelling, no open wound or discoloration, and neurovascular examination was intact; a capillary refill was less than two seconds. X-rays were done for him in the ED with no fracture seen, so they kept him on below elbow splint and arranged orthopedic clinic appointment.

**Figure 1:**
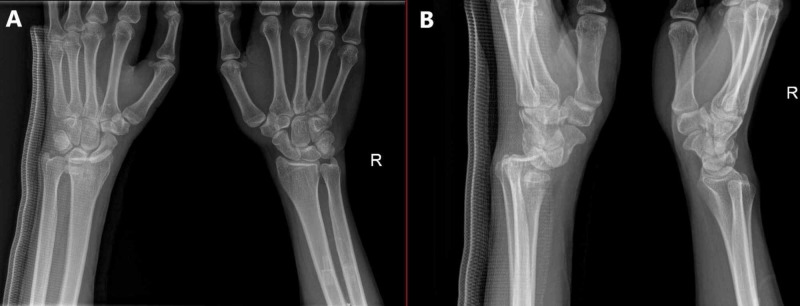
X-ray of the left wrist (injured) and contralateral wrist. A) Anteroposterior (AP) view ; B) Lateral view

**Figure 2:**
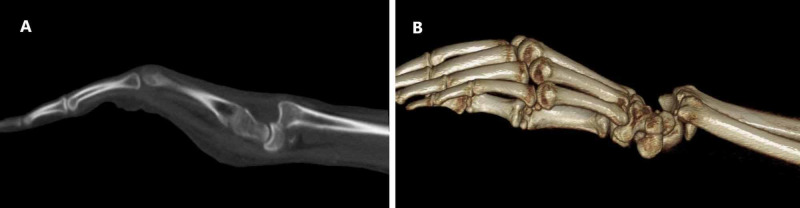
Preoperative CT images of the left wrist. A) Standard CT image; B) 3D CT image

Two days later, when we were reviewing the X-ray imaging in our department, we noticed a missed diagnosis of left wrist joint complete volar RCD, and the patient was called for urgent surgical treatment (Figure 1). All preoperative laboratory investigations and left wrist CT were done on presentation, which confirmed the finding (Figure 2).

**Figure 3:**
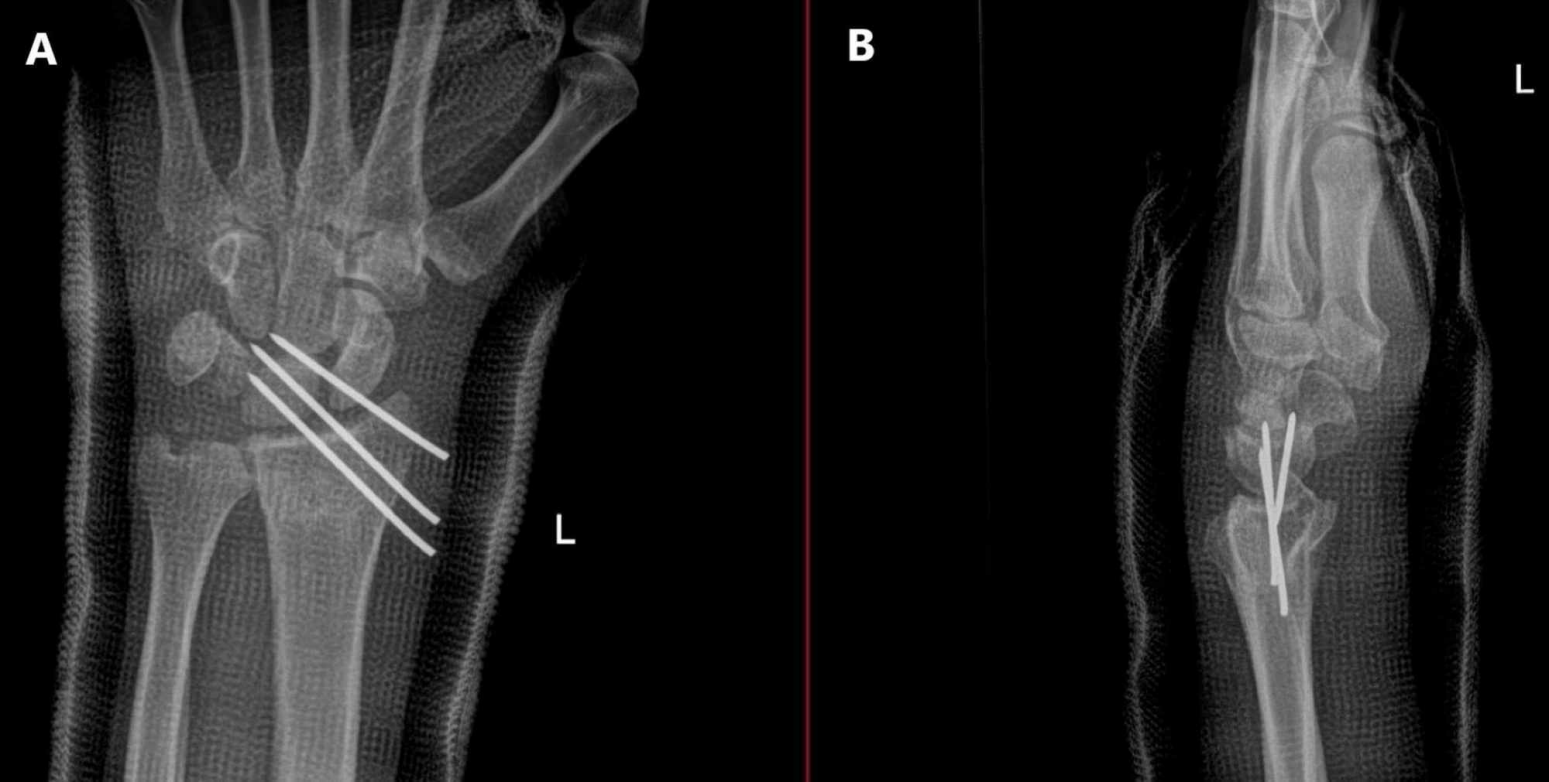
Intraoperative X-ray of the left wrist. A) Anteroposterior (AP) view; B) Lateral view

**Figure 4:**
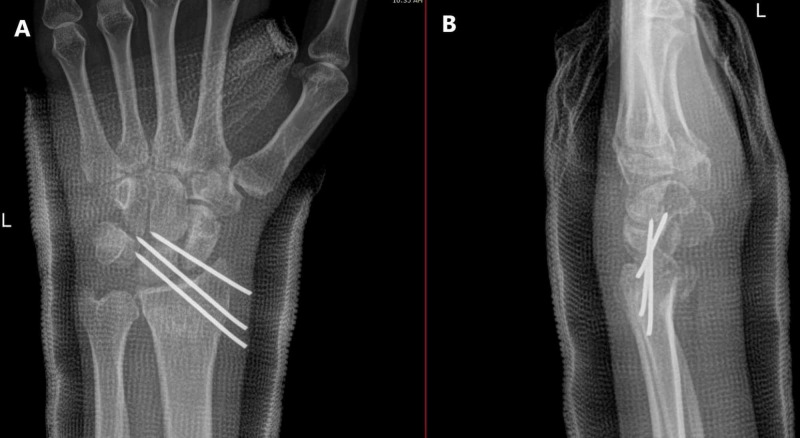
Twelve days postoperation, left wrist. A) Anteroposterior (AP) view; B) Lateral view

The next day (day ten since the initial injury), we did a close reduction by simple traction, counter traction, with two thumbs pushing volary to reduce the dislocated radiocarpal joint.. Then three lateral to medial parallel percutaneous Kirschner wires pinning of the left radiocarpal joint were made under general anesthesia using fluoroscopic guidance; then below elbow cast was applied (Figure 3). The patient came to the clinic 12 days postoperation, was doing well without new complaints, the distal neurovascular exam was intact, and new X-ray imaging was good (Figure 4).

**Figure 5:**
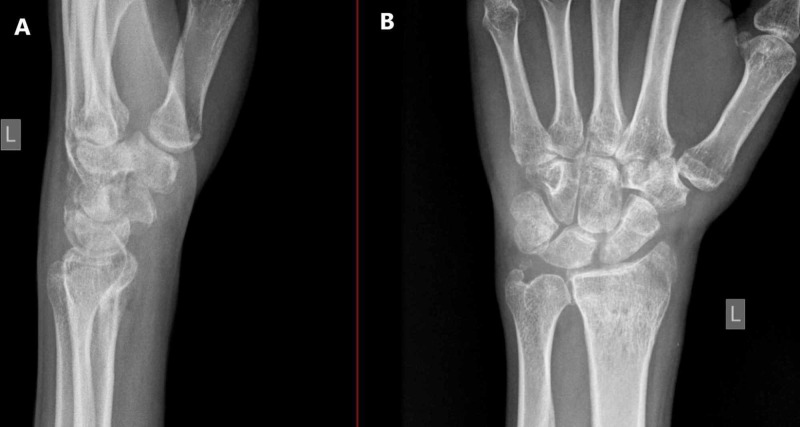
X-ray of the left wrist post wires removal surgery. A) Lateral view; B) anteroposterior (AP) view

Five weeks postoperation, the cast was removed to examine the joint and skin, and the wrist joint condition improved. Still, there was mild swelling without signs of infection. The cast was converted to thumb spica for the next three weeks. Eight weeks postoperation, the patient was admitted for wires removal under local anesthesia, and then wrist brace was applied (Figure 5).

**Figure 6:**
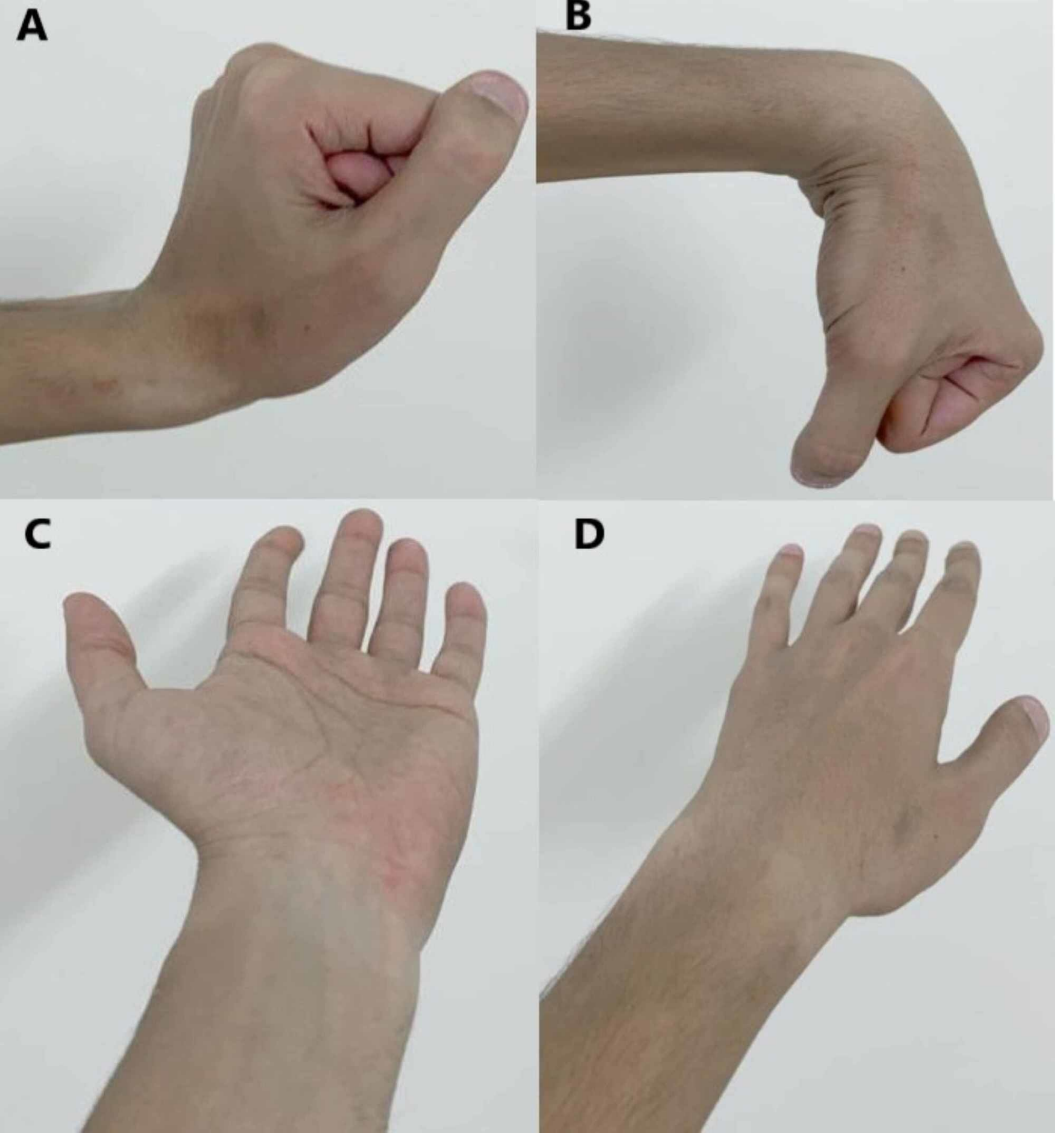
Left wrist range of motion upon discharge. A) Extension; B) Flexion; C) Supination; D) Pronation

**Figure 7:**
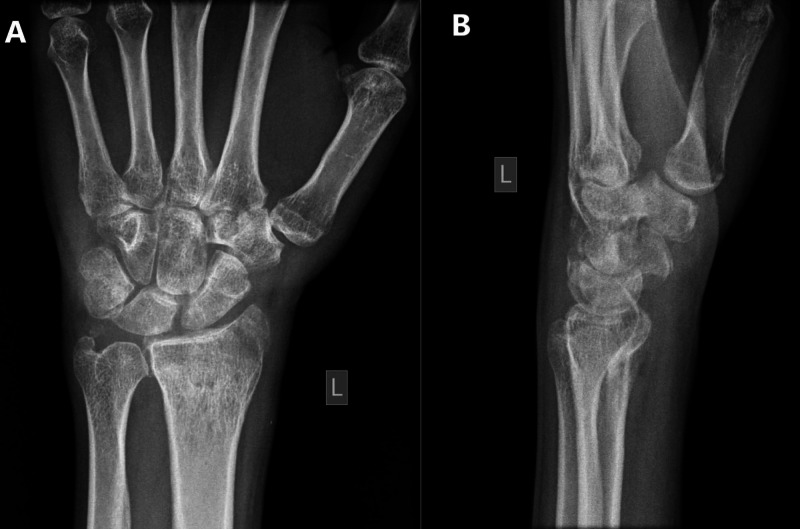
X-ray of the left wrist before discharge. A) Anteroposterior (AP) view; B) Lateral view

We referred the patient to occupational therapy to improve the range of motion and muscle strengthening. During clinic follow-up, the painless range of motion was improved as the extension was from zero to 20-degree, flexion from zero to 30-degree, full supination, pronation, and muscle power four over five. The patient was satisfied with the results and started to use his hand normally. After three months follow-up, the patient presented without any complication or a new issue with a good range of motion and was discharged from Orthopedic service after evaluating his recent X-ray images (Figures 6-7).

 

## Discussion

Radiocarpal dislocations are uncommon injuries of the wrist that are usually caused after a severe injury to the carpus leading to intercarpal supination and disruption of the volar extrinsic radiocarpal ligaments [1-2]. RCDs are reported in individuals who are victims of high-velocity trauma such as road traffic accidents, fall from heights, industrial accidents, or violence and could involve bone, ligament, and soft-tissue disruption [5, 9-10]. 

Radiocarpal dislocation is classified into two categories based on the guidance for treatment. Type 1 refers to dislocation or a fracture-dislocation involving fracture of the tip of the radial styloid process, whereas type 2 RCD corresponds to dislocation with a fracture of a larger fragment of styloid of the radius passing through the scaphoid facet [5]. In both types, it is common to find median nerve compression, and patients often experience neurapraxia [11]. Thus, the surgical treatment of RCDs includes ligamentous repair combined with percutaneous Kirschner wire pinning and/or distraction-plating with or without external fixation using either open or closed reduction method [3,12-14]. It is often difficult to maintain normal radiocarpal relationships despite the belligerent treatment undertaken [15]. When the above procedures fail, a reinstating surgical immobilization such as radioscaphoid, radiolunate, or total wrist arthrodesis is the next choice [12, 16-17]. 

Another consideration when performing radiographic investigation is that an X-ray of the contralateral wrist should also be obtained in a single film or separately, which will help to distinguish between congenital laxity of wrist ligaments and instability following the trauma [18]. A CT scan is the recommended radiographic investigation in difficult RCD cases to evaluate the wrist and scaphoid that will help rule out avulsion fractures and associated osseous trauma [9-10, 19]. As in our case, it was difficult during the first time to rule out the injury in the normal X-ray; a CT scan helped to evaluate and confirm the RCD. MRI and wrist arthroscopy are also useful for identifying intra-articular soft tissue injuries in such cases [4, 11]. 

The management of RCD is still debatable, and various authors have suggested different modalities based on the circumstances and also on the severity of the injury. Some authors have claimed success with closed reduction and immobilization, while some others have suggested open reduction with fixation [2, 4, 12, 14, 20]. Some authors recommend external fixation, as it is useful not only for reducing the dislocation and associated fractures, but also helps in keeping the repaired ligaments under optimal tension [7]. It is often suggested that postoperative immobilization with a long arm cast should be done for a minimum of three weeks and a maximum of 10 weeks depending upon the time taken to heal the ligaments [6-8]. Reports show that there could be associated complications with RCDs such as neurovascular injuries, compartment syndrome, and carpal tunnel syndrome [4]. In this case, the patient was satisfied with the functional outcomes and performed his normal activities. Early surgical treatment involving the restoration and stabilization of the radiocarpal ligament is essential for achieving good outcomes [9].

## Conclusions

Volar RCDs are rare and are not a frequently reported injury. They are often difficult to diagnose using a conventional X-ray and so a CT scan is often recommended to rule out avulsion fractures and associated osseous trauma. Open reduction or closed reduction with K-wire pinning is the usual surgical treatment followed depending upon the severity of the injury.
